# Factors associated with outcome of endovascular treatment of iliac occlusive disease: a single-center experience

**DOI:** 10.1590/1677-5449.003817

**Published:** 2018

**Authors:** Rafael de Athayde Soares, Marcelo Fernando Matielo, Francisco Cardoso Brochado-Neto, Marcus Vinícius Martins Cury, Veridiana Borges Costa, Maria Clara Pereira Sanjuan, Christiano Stchelkunoff Pecego, Roberto Sacilotto

**Affiliations:** 1 Serviço de Cirurgia Vascular e Endovascular, Hospital do Servidor Público Estadual de São Paulo, São Paulo, SP, Brasil.

**Keywords:** endovascular treatment, iliac occlusive disease, critical limb ischemia, incapacitating claudication, major amputation, tratamento endovascular, doença oclusiva líaca, isquemia crítica, claudicação limitante, amputação maior

## Abstract

**Background:**

Endovascular treatment (ET) of iliac occlusive disease (IOD) is well established in literature. Use of stents in IOD has achieved long-term limb salvage and patency rates similar to those of open surgery, with lower morbidity and mortality rates.

**Objectives:**

To report the long-term outcomes, particularly limb salvage and patency rates, of ET for IOD and the factors associated with these outcomes.

**Methods:**

This retrospective cohort study included patients with IOD who underwent iliac angioplasty (IA), between January 2009 and January 2015. Patients with critical limb ischemia or incapacitating claudication were included.

**Results:**

In total, 48 IA procedures were performed in 46 patients, with an initial technical success rate of 95.83%. Failure occurred in two patients, who were excluded, leaving 44 patients and 46 IA. The primary patency, secondary patency, limb salvage, and survival rates at 1200 days were 88%, 95.3%, 86.3%, and 69.9%, respectively. Univariate and multivariate Cox regression revealed that the primary patency rate was significantly worse in patients with TASC type C/D than in patients with TASC type A/B (p = 0.044). Analysis of factors associated with major amputation using Cox regression showed that the rate of limb loss was greater in patients with TASC type C/D (p = 0.043). Male gender was associated with reduced survival (p = 0.011).

**Conclusions:**

TASC type C/D was associated with a higher number of reinterventions and with worse limb loss and primary patency rates. Male gender was associated with a worse survival rate after ET of IOD.

## Resumo

**Contexto:**

O tratamento endovascular da doença oclusiva ilíaca (DOI) é bem estabelecido. O uso de stents nas angioplastias ilíacas (AI) alcançou estimativas de salvamento de membro e perviedade similares às de cirurgias abertas, porém com menor morbimortalidade.

**Objetivos:**

Demonstrar os desfechos clínicos a longo prazo, principalmente as estimativas de salvamento de membro (ESM) e perviedade, do tratamento endovascular da DOI e os fatores associados.

**Método:**

Estudo de coorte retrospectiva e consecutiva incluindo pacientes com DOI e isquemia crítica ou claudicação limitante submetidos a AI entre janeiro de 2009 a janeiro de 2015.

**Resultados:**

Foram realizadas 48 AI em 46 pacientes, com uma taxa de sucesso técnico inicial de 95,83%. Ocorreu falha técnica em dois pacientes, os quais foram excluídos da análise, restando 44 pacientes e 46 AI. As estimativas de perviedade primária, perviedade secundária, ESM e sobrevida aos 1.200 dias foram de 88%, 95,3%, 86,3% e 69,9%, respectivamente. A regressão de Cox univariada e multivariada revelou que a perviedade primária foi pior em pacientes com classificação TASC C/D do que em pacientes TASC A/B (p = 0,044). Quando analisamos os fatores associados à amputação maior, verificou-se que lesões TASC tipo C/D (p = 0,043) apresentaram piores resultados. O sexo masculino foi associado com sobrevida reduzida (p = 0,011).

**Conclusões:**

Classificação TASC tipo C/D foi associada a um maior número de reintervenções, maior perda de membro e piores estimativas de perviedade primária. O sexo masculino foi associado a uma pior sobrevida.

## INTRODUCTION

 Treatment of iliac occlusive disease (IOD) has undergone substantial changes in the last few years. Previously, most treatment guidelines recommended endovascular intervention for single, short, and focal lesions, and open surgery for extensive IOD. However, with recent improvements in technology and endovascular techniques, guidelines are now advocating endovascular treatment instead of open surgery as the primary treatment for focal or extensive disease. 1 ^,^ 2 Use of stents in iliac angioplasty has also achieved long-term limb salvage and patency rates similar to those of open surgery, but with much lower morbidity and mortality rates. 3

 The purpose of this paper is to report the long-term outcomes, focusing on limb salvage and patency, of endovascular treatment for IOD and the factors associated with these outcomes in a single center. 

## METHODS

 The study was approved by the research ethics committee at Hospital do Servidor Público Estadual de São Paulo, SP, Brazil. This retrospective cohort study included consecutive patients with IOD who underwent iliac angioplasty at Serviço de Cirurgia Vascular e Endovascular, Hospital do Servidor Público Estadual de São Paulo, between January 2009 and January 2015. Patient data were collected from the hospital’s database using Microsoft Access® software (Microsoft, Seattle, WA). Data obtained included the patient’s general and demographic characteristics and information recorded during outpatient follow-up visits. The patient’s hospital records were also consulted if necessary. Details of the surgical procedures were retrieved from the database and the patient’s medical records. In our department, all patients undergo cardiac evaluation by a specialist to assess their cardiac risk based on the guidelines for perioperative cardiovascular evaluation and care in non-cardiac surgery published by the American Heart Association. 4

 Patients with critical limb ischemia or incapacitating claudication who underwent iliac angioplasty during the index period were eligible for the study. Patients who experienced an initial technical failure were excluded from the study. The arteriographic procedures in all patients were re-evaluated to ensure the protocols were accurately reported. After reviewing the arteriographs, the patients were classified according to established protocols, including the Trans-Atlantic Inter-Society Consensus (TASC) II classification, 5 ^,^ 6 according to the diameter and length of the catheter balloons and stents, and according to other factors related to endovascular treatment. 

 In our department, we prefer to use bare-metal stents for iliac angioplasty. Patients are given 300 mg of clopidogrel as a loading dose immediately after the procedure, and continue taking 75 mg of clopidogrel per day for 6 months after surgery and aspirin (100 mg/day) for life. This dose of aspirin was administered to all patients unless there were contraindications. All patients receive some type of statin before and after surgery. The indications for angioplasty were based on prior reports, such as TASC II. 5 ^,^ 6

 Initial technical success of iliac angioplasty was defined as residual stenosis of ≤ 30%, no dissection after the procedure, and prompt restoration of blood flow in the previously stenotic or occluded artery. Procedures such as debridement and minor amputations were also performed during hospitalization, if necessary. 

 All the patients were followed-up via outpatient visits at the following times after discharge: 15 days, 1 month, 3 months, 6 months, 12 months, and every 6 months thereafter. The following information was recorded at each visit: pulse palpation, ankle–brachial index (ABI), and symptoms. Whenever possible, we also performed surveillance with arterial duplex ultrasonography at 1, 3, 6, and 12 months after surgery, and every 6 months thereafter. If any clinical or sonographic changes were noted, the case was discussed at a departmental meeting to assess whether re-intervention was required. Occlusion of the treated artery was defined, based on duplex ultrasonography, as no flow, segmental, or complete. 

 The primary outcome variable was limb salvage. Major amputation was defined as amputation proximal to the ankle. The secondary outcome variables were patency, survival, and operative mortality. 

 Statistical analyses were performed with SPSS 15.0 for Windows® (SPSS Inc., Chicago, IL). Percentages of patients and descriptive statistics were calculated. The χ ^2^ test and Student’s *t* test were used to compare univariate data. Survival curves were constructed for estimated limb salvage, patency, and survival rates using the Kaplan–Meier method. Cox regression was used in univariate and multivariate analyses. In all analyses, *p*-values of < 0.05 were considered statistically significant. 

## RESULTS

 In total, 48 iliac angioplasties were performed in 46 patients, with an initial technical success rate of 95.83% of patients. The mean ± standard deviation (SD) follow-up time was 1200±680.84 days. Analyses were performed at 1200 days. Technical failure occurred in two patients, and was due to failure to progress a 0.035” guidewire through the lesion. These patients were excluded from the analysis, leaving 44 patients and 46 aortoiliac angioplasties. The excluded patients were treated with open revascularization procedures, one with aortobifemoral bypass and the other with iliofemoral bypass. 

 The general characteristics of the patients are shown in Table 1 and perioperative data are listed in Table 2 . The indication for revascularization was critical limb ischemia in most of the patients (79.53%) ( Table 2 ). The perioperative mortality rate was 4.5% ( Table 2 ). The endovascular procedures are summarized in Table 3 . The left limb was treated in the majority (60.8%) of patients. The ipsilateral and contralateral retrograde femoral arteries were the most common puncture sites (41.3% and 39.1% of patients, respectively). The common iliac artery was the most commonly treated artery (in 84.64% of patients). Bare metal stents were used in nearly all (95.62%) of the patients, while an expandable balloon stent was used in 56.52% of patients. When we classified the arteriographic lesions according to the TASC II classification, type B lesions were the most common (in 45.6% of patients). 

**Table 1:** General characteristics.

**Variable**	**Value**
Age, years	66±9.1
Female	24 (54.5%)
Concomitant diseases	
Hypertension	40 (90.9%)
Diabetes mellitus	23 (52.3%)
Heart disease	6 (13.6%)
Chronic kidney failure	7 (15.9%)
Tobacco use	35 (79.5%)

Data are expressed as the mean ± standard deviation or number (percentage) of patients.

**Table 2:** Perioperative data.

**Variable**	**Value**
Elevated cardiac risk, yes	13 (29.5)
Infection	20 (45.5)
Perioperative mortality rate	2 (4.5)
Incapacitating claudication	11 (25)
Pain at rest	7 (15.9)
Trophic Lesion	28 (63.6)

Data are expressed as the number (percentage) of patients.

**Table 3:** Endovascular procedures.

**Variable**	**Value**
Limb treated	
Right	18 (39.1)
Left	28 (60.8)
Artery puncture site	
Ipsilateral retrograde femoral artery	19 (41.3)
Contralateral retrograde femoral artery	18 (39.1)
Brachial	9 (19.5)
Arteries treated	
Common iliac	25 (54.3)
External iliac	7 (15.2)
Common iliac + external iliac	12 (26)
Aorta + common iliac	2 (4.3)
Stenting	44 (95.6)
Stent type	
Self-expanding	18 (39.1)
Balloon-expanding	26 (56.5)
TASC lesion type	
A	17 (36.9)
B	21 (45.6)
C	4 (8.7)
D	4 (8.7)

Data are expressed as the number (percentage) of patients.

 The mean ABI was greater after surgery than before surgery (0.65 vs. 0.52, p = 0.01). Seven (15.9%) patients required reintervention, with endovascular surgery in four patients (57.1%), aortobifemoral bypass surgery in two patients (28.6%), and femorofemoral bypass in one (14.3%). Multivariable Cox regression revealed that TASC type C/D lesions were associated with a higher number of reinterventions (p = 0.001), as shown in Table 4 . 

**Table 4:** Cox regression analysis of factors associated with reintervention.

**Variable**	**Univariate analysis**				**Multivariate analysis**			
	**B**	**HR**	**95%CI**	***p*-value**	**B**	**HR**	**95% CI**	***p*-value**
Diabetes mellitus	0.403	1.49	0.411-5.455	0.541	0.207	1.23	0.293-5.168	0.777
Chronic kidney failure	13.355	5.00	0.419-6306	0.419	13.167	9.00	0.411-5.455	0.984
Tobacco use	−0.692	0.50	0.109-2.297	0.373	0.664	1.94	0.247-15.244	0.528
High surgical risk	10.085	1.00	0.112-1.488	0.345	6.981	0.54	0.135-2.198	0.080
TASC type C/D	11.527	1.41	0.388-5.188	0.001	2.064	7.87	1.004-61.763	0.050
Iliac segment treated †	0.319	1.36	0.361-5.163	0.572	−0.132	0.87	0.132-5.862	0.891
Indication for revascularization ‡	0.892	0.53	0.334-3.233	0.345	0.127	1.13	0.114-11.316	0.914

B = coefficient; HR = hazard ratio; CI = confidence interval.

† Iliac segment treated: common iliac artery + external iliac artery vs. common iliac artery;

‡ Indication for revascularization: critical limb ischemia vs. claudication.

 Univariate and multivariate Cox regression showed that the primary patency rate was significantly worse in patients with TASC type C/D lesions than in patients with TASC type A/B lesions (multivariate p = 0.044). Other factors, such as tobacco use, presence of diabetes mellitus, presence of chronic kidney failure, and the iliac segment treated were not associated with the primary patency rate, as shown in Table 5 . 

**Table 5:** Cox regression analysis of factors associated with primary patency.

**Variable**	**Univariate analysis**				**Multivariate analysis**			
	**B**	**HR**	**95%CI**	***p*-value**	**B**	**HR**	**95%CI**	***p*-value**
Chronic kidney failure	1.041	4.00	0.419-6306	0.307	11.156	6.98	0.316-38.511	0.979
Tobacco use	2.214	1.45	0.109-2.297	0.966	−13.410	3.48	0.316-38.511	0.308
TASC type C/D	18.521	10.42	0.388-5.188	0.000	2.866	17.56	1.075-286.992	0.044
Iliac segment treated †	1.756	1.36	0.221-1.501	0.185	2.840	17.11	0.372-786.351	0.146
Diabetes mellitus	0.016	0.81	0.016-4.684	0.900	−1.310	0.27	0.016-4.864	0.368

B = coefficient; HR = hazard ratio; CI = confidence interval.

† Iliac segment treated: common iliac artery + external iliac artery vs. common iliac artery.

 When we analyzed the factors associated with major amputation using Cox regression, we found the rate of limb loss was greatest in patients with TASC type C/D lesions (p = 0.043). The other factors included in the analysis (gender, TASC type A/B lesions, iliac segment treated, presence of chronic kidney failure, and tobacco use) were not predictors of the rate of limb loss ( Table 6 ). 

**Table 6:** Cox regression analysis of factors associated with limb salvage.

**Variable**	**Univariate analysis**				**Multivariate analysis**			
	**B**	**HR**	**95%CI**	***p*-value**	**B**	**HR**	**95%CI**	***p*-value**
Chronic kidney failure	13.320	6.00	0.419-6306	0.233	11.634	7.00	0.316-38.511	0.982
Tobacco use	0.021	0.50	0.109-2.297	0.589	1.249	3.48	0.316-38.511	0.308
TASC type C/D	9.098	10.42	0.388-5.188	0.010	2.344	10.42	1.007-100.818	0.043
Iliac segment treated †	2.657	1.36	0.221-1.501	0.572	0.103	2.69	0.538-13.494	0.228

B, coefficient; HR, hazard ratio; CI, confidence interval.

† Iliac segment treated: common iliac artery + external iliac artery vs. common iliac artery.

 Finally, we analyzed the factors associated with overall survival using univariate and multivariate Cox regression. These analyses included the factors gender; presence of hypertension, diabetes mellitus, heart disease, or chronic kidney failure; tobacco use; elevated cardiac risk; and TASC type. Of these factors, only male gender was associated with reduced survival (p = 0.011) ( Table 7 ). 

**Table 7:** Cox regression analysis of factors associated with overall survival.

**Variable**	**Univariate analysis**				**Multivariate analysis**			
	**B**	**HR**	**95%CI**	***p*-value**	**B**	**HR**	**95%CI**	***p*-value**
Diabetes mellitus	−0.482	0.61	0.175-2.176	0.453	0.207	−0.482	0.175-2.176	0.453
Chronic kidney failure	−0.693	0.50	0.095-2.626	0.419	−0.693	0.50	0.095-2.626	0.419
Tobacco use	0.276	1.31	0.314-5.536	0.706	0.276	1.31	0.314-5.536	0.706
High surgical risk	−0.292	0.74	0.072-7.698	0.806	6.981	0.54	0.135-2.198	0.080
TASC type C/D	−0.517	0.59	0.388-5.188	0.519	−0.517	0.59	0.388-5.188	0.519
Male gender	−2.324	0.09	0.016-0.590	0.011	−2.324	0.09	0.016-0.590	0.011
Heart disease	-0.371	1.44	0.211-9.933	0.706	−0.371	1.44	0.211-9.933	0.706

B = coefficient; HR = hazard ratio; CI = confidence interval.

**Figure 1:**
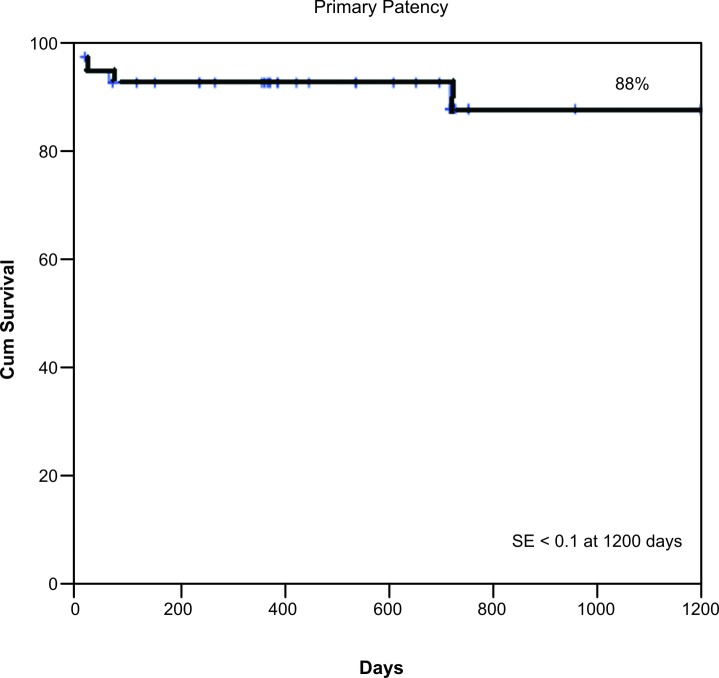
Kaplan–Meier analysis of the primary patency rate. The primary patency rate at 1200 days was 88% and the standard error was < 10%.

**Figure 2:**
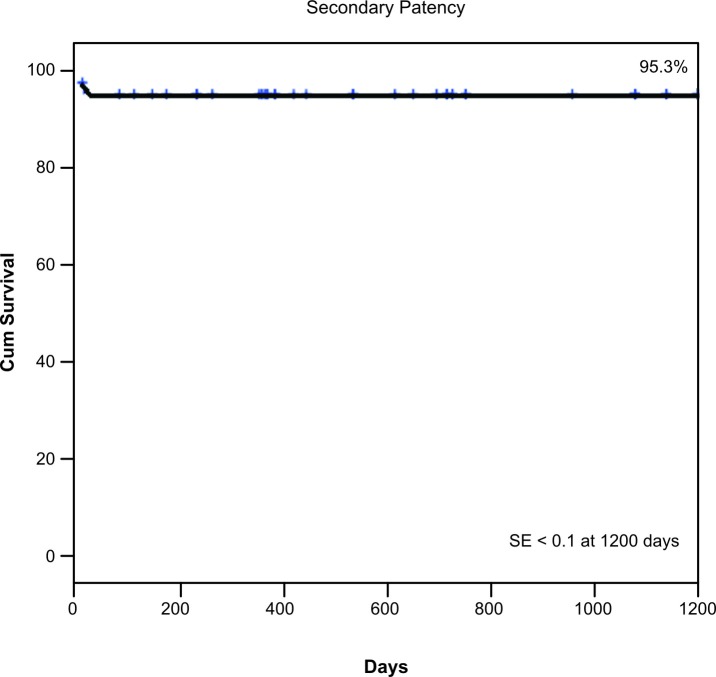
Kaplan–Meier analysis of the secondary patency rate. The secondary patency rate at 1200 days was 95.3% and the standard error was < 10%.

**Figure 3:**
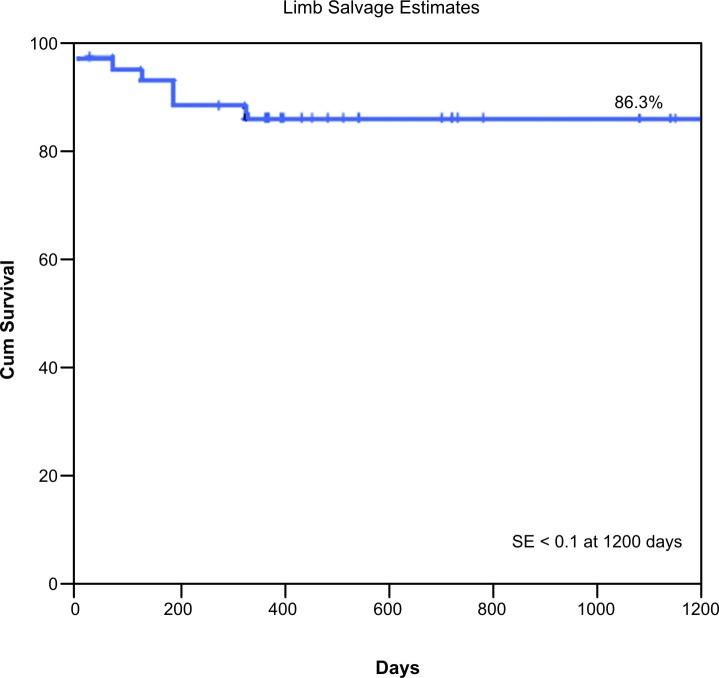
Kaplan–Meier analysis of the limb salvage rate. The limb salvage rate at 1200 days was 86.3% and the standard error was < 10%.

**Figure 4:**
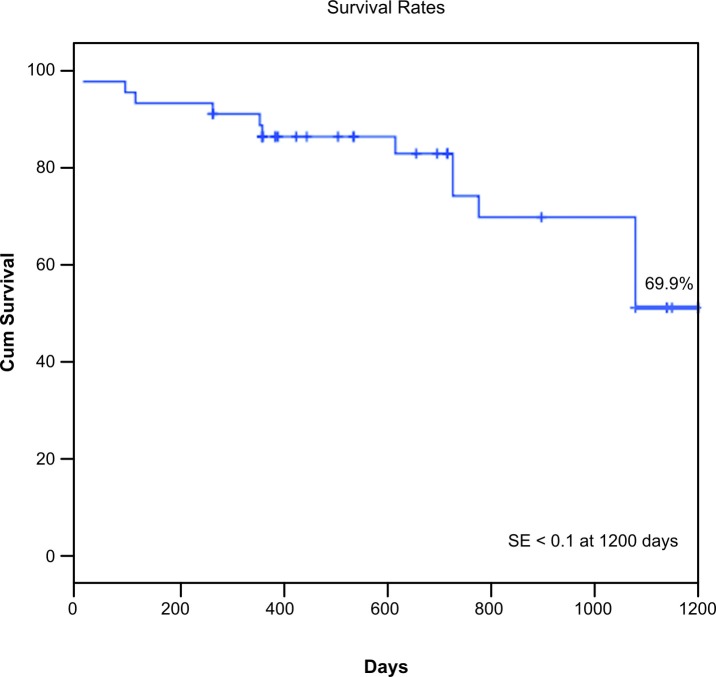
Kaplan–Meier analysis of overall survival. The survival rate at 1200 days was 69.9% and the standard error was < 10%.

 The estimated primary patency, secondary patency, limb salvage, and survival rates at 1200 days were 88% ( Figure 1 ), 95.3% ( Figure 2 ), 86.3% ( Figure 3 ), and 69.9% ( Figure 4 ), respectively. 

## DISCUSSION

 Although this was a retrospective study, the factors associated with worse outcomes of endovascular treatment of AIOD were similar to the factors reported in prior studies. Kudo et al. 7 analyzed the long-term outcomes and predictors of outcome after iliac angioplasty in 151 patients. In that study, the cumulative primary patency rates at 1, 3, and 5 years were 76%, 59%, and 49%, respectively. The cumulative assisted primary patency, secondary patency, and limb salvage rates at 7 years were 98%, 99% and 93%, respectively. They reported that significant independent predictors for adverse outcomes were smoking history (p = 0.0074), TASC type C/D lesions (p = 0.0001), and stenosis of the ipsilateral superficial femoral artery (p = 0.0002) for the primary patency rates. Meanwhile, chronic kidney failure with hemodialysis (p = 0.014), ulcer/gangrene as an indication for percutaneous transluminal angioplasty (p < 0.0001), and stenosis of the ipsilateral superficial femoral artery (p = 0.034) were associated with adverse outcomes. 

 Kavaliauskiene et al. 8 recently evaluated the risk factors for adverse outcomes of iliac artery stenting. In their prospective study, they evaluated 62 iliac endovascular procedures in 54 patients. The primary patency rate was 79.9% at 2 years. They reported that stent location in the common iliac artery and external iliac artery, and poor runoff were independent predictors of decreased primary stent patency. In our study, we showed that presence of TASC type C/D lesions was an independent predictor of limb loss (p = 0.043) and of reintervention (p = 0.001) following iliac stenting. 

 Despite the high prevalence of patients with elevated cardiac risk (29.5%), the overall perioperative mortality rate in our study was 4.3%, which is satisfactory. However, the survival rate at 1200 days was 69.9%. When we analyzed the factors associated with survival using Cox regression, only male gender was associated with reduced survival, out of all the factors analyzed (gender; TASC II classification; presence of heart disease, diabetes mellitus, or chronic kidney failure; tobacco use; and cardiac risk). Our estimated survival rate and perioperative mortality rate are similar to those reported in other studies. For example, in an analysis by Timaran et al. 9 of 136 endovascular iliac procedures, the perioperative mortality rate was 0.7% and the 5-year survival rate was 78%. However, the only independent predictors of decreased long-term survival in multivariate analyses were smoking history (relative risk: 3.9; 95% confidence interval [CI] 1.9-8.4; p < 0.001) and hyperlipidemia (relative risk: 2.7; 95%CI 1.3-5.7; p = 0.007). Similarly, Kasemi et al. 10 examined the outcomes for up to 7 years after endovascular treatment of AIOD in 22 patients with TASC type D lesions. The perioperative mortality rate in their study was 4.5%. 

 Jongkind et al. 11 performed a systematic review of 19 nonrandomized cohort studies involving a total of 1711 patients who underwent endovascular treatment of extensive IOD. The mortality rate ranged from 1.2 to 6.7%. The 4- or 5-year primary patency rate ranged from 60 to 86% and secondary patency rates ranged from 80 to 98%. In the present study, the estimated primary patency, secondary patency, limb salvage, and survival rates at 1200 days were 88%, 95.3%, 86.3%, and 69.9%, respectively. These rates are similar to those reported in the literature described above. 

 Ye et al. 12 assessed early and late outcomes of endovascular treatment in patients with TASC type C/D aortoiliac lesions in a systematic review. The technical success and 12-month primary patency rates were 94.2% (95%CI 91.8-95.9) and 92.1% (95%CI 89.0-94.3), respectively, for primary stenting and were 88.0% (95%CI 67.9-96.2) and 82.9% (95%CI 72.2-90.0), respectively, for selective stenting (used only in cases of dissection or recoil higher than 30%). They concluded that the long-term primary patency rate was significantly better in patients who underwent primary stenting than in patients who underwent selective stenting. In our study, stents were used in the nearly all of the patients (96%). This might help explain the moderate estimated primary patency, secondary function, and limb salvage rates in our study. 

 In our study, the iliac segment subjected to endovascular treatment (common iliac artery and external iliac artery vs. common iliac artery) did not influence the limb salvage, reintervention, or primary patency rates. Similarly, Danczyk et al. 13 compared the outcomes between multiple ipsilateral iliac artery stents and isolated iliac artery stents. They found that survival, reintervention-free survival, late open conversion-free survival, and amputation-free survival were similar between the patient groups (all p > 0.05). Combined stenting of the common and external iliac arteries was not a predictor of death, reintervention, late open conversion, or amputation. 

 The presence of TASC type C/D lesions was the only factor independently associated with a higher rate of reinterventions in this study based on the results of univariate and multivariate Cox regression analysis. Kudo et al. 14 examined the outcomes of percutaneous transluminal angioplasty for the treatment of critical limb ischemia in 111 patients. Their analysis included 12 possible predictors of outcomes and they concluded that hypertension, multiple segment lesions, more distal lesions, and TASC type D lesions were significant and independent risk factors for the outcomes (all p < 0.05; univariate log-rank test and multivariate Cox regression analysis). In our study, multivariate Cox regression analyses revealed that the presence of TASC type C/D lesions was associated with worse primary patency and limb salvage rates and a higher rate of reinterventions, and that male gender was associated with a worse survival rate. 

 According to Mwipatayi et al., 15 covered stents have better patency rates in IOD endovascular treatment, when compared to bare-metal stents at 5 years, especially in calcified aortic bifurcations. However, the choice of stent does not affect the rate of major amputations. The present study did not aim to compare differences between stents. 

 This study has some important limitations to report. In particular, it was a retrospective study involving analysis of consecutive data collected from medical records and our institutional database. These limitations could be addressed by a prospective study. Although the statistical method employed in this study was appropriate, the possibility of type II error (beta) may nevertheless be considerable, due to the small number of patients classified as TASC C/D. 

## CONCLUSION

 Based on the results of this study, we conclude that endovascular treatment of IOD is safe, is associated with satisfactory and reproducible long-term outcomes, and has a low perioperative mortality rate, particularly in patients with TASC type A/B lesions. Presence of TASC type C/D lesions was associated with a higher number of reinterventions and with worse limb loss and primary patency rates, consistent with earlier studies. We also showed that male gender was associated with a worse survival rate after endovascular treatment of IOD. 
